# Secondary overtriage of trauma patients in a trauma center: frequency and associated factors

**DOI:** 10.5249/jivr.v15i1.1701

**Published:** 2023-01

**Authors:** Shandiz Moslehi, Zahra Haghdoust, Gholamreza Masoumi, Enayatollah Homaie Rad, Fatemeh Nouri, Leila Kouchakinejad-Eramsadati, Sakineh-Khatoun Ranjkesh Ziabari, Alireza Azizpour

**Affiliations:** ^*a*^ Health Management and Economics Research Center, Health Management Research Institute, Iran University of Medical Sciences, Tehran, Iran.; ^*b*^ Guilan Road Trauma Research Center, Guilan University of Medical Sciences, Guilan, Iran.; ^*c*^ Department of Health in Emergencies and Disasters, School of Health Management and Information Sciences, Iran University of Medical Sciences, Tehran, Iran.; ^*d*^ Social Determinants of Health Research Center, Poursina Hospital, Guilan University of Medical Sciences, Guilan, Iran.; ^*e*^ Department of Health in Emergencies and Disasters, School of Public Health and Safety, Shahid Beheshti University of Medical Sciences, Tehran, Iran.; ^*f*^ Department of Health in Emergencies and Disasters, School of Public Health, Tehran University of Medical Sciences, Tehran, Iran.; ^*g*^ Poursina Hospital, Guilan University of Medical Sciences, Rasht, Iran.

**Keywords:** Transfer, Injury Severity Score, Hospital, Education

## Abstract

**Background:**

Secondary overtriage (SO) is the unnecessary transfer of traumatic patients between facilities, which causes the waste of the resources of the trauma centers and imposes extra costs on patients and caregivers. This study aimed to determine the frequency of secondary overtriage and patient-level, clinical, and hospital factors leading to secondary overtriage.

**Methods:**

This cross-sectional study evaluated the data of all trauma patients who were transferred to a high-level trauma center in Guilan between 2016 and 2020. The patients with SO were characterized as those transported to a trauma center with an injury severity score ≤15 and discharged alive within 48 hours without undergoing surgical procedure. Secondary overtriage and appropriate transmissions were analyzed using descriptive statistics. Multivariate logistic regression was used to identify the relationship between SO and patient-level, clinical, and hospital factors.

**Results:**

Out of 3342 transferred trauma patients, 3091(92.49%) had the inclusion criteria. The rate of SO was 25.68 % (794). These patients were younger (median 34 versus 36), with 253 women and 541 men. The highest SO belonged to spine injuries (109, 38.2%) (P less than 0.05). In both secondary overtriaged and appropriately triaged patients, the main cause of transmission was the shortage of neurosurgeons (741, 93.3% and 1780, 77.5%) (P less than 0.05). At the patient level, sex (OR 0.632, 95%CI 0.480-0.832) and at the clinical level, injured body region (specifically spine injury (OR 2.233, 95%CI 1.472-3.388), the reason for transfer (OR 2.158, 95%CI 1.185-3.927), injury severity score (OR 0.655, 95%CI 0.0615-0.697) and length of stay (OR 0.368, 95%CI 0.317-0.428) had a significant relationship with secondary overtriage.

**Conclusions:**

About a quarter of transferred traumatic patients were identified as secondary overtriage. Continuous medical education, recruiting trained staff, improving the transfer protocols, extending collaborations between low-level/non-trauma and high-level trauma centers and using telemedicine can provide medical staff with more efficient guidance on transfer decision.

## Introduction

Traumatic patients should be transferred to appropriate trauma centers in the golden time according to their medical needs. The overriding principle is that patients with more severe trauma should be transferred to centers with higher levels of care, whereas patients with milder trauma can receive care at the hospital where they are initially admitted or a lower-level trauma center.^1,2^ However, the results of studies have shown that in most cases, patients with minor injuries who do not need advanced trauma care are transferred to high-level trauma centers (HTCs) due to the lack of resources in low-level and non-trauma centers (LNTCs).^3^ Consequently, a percentage of patients are discharged within 48 hours of arrival in the trauma center without undergoing any surgical procedure. This situation is defined as unnecessary transfer or secondary overtriage (SO).^4^ Evidence has shown that over a third of traumatic patients transferred to the emergency department (ED) were SO cases who were discharged without admission, observation, or undergoing surgical procedures.^5,6^

Prior studies have reported several reasons for SO, including age, sex, night shift admission, limited access to the surgeon, limited blood products, lack of imaging equipment at transferring hospital,^2,4^ type of insurance,^7^ spinal cord injury,^8,9^ and traumatic brain injuries.^10,11^ However, none of the previous research has conclusively claimed that transferring patients with minor injuries to HTCs would improve their survival.^4,6^ Even sometimes, SO can have adverse consequences for the health system, the hospital, and the patient; and waste the limited resources of HTCs. It causes overcrowding and shifting resources from severely traumatic patients who need care in a well-equipped trauma center to patients with minor injuries,^12^ in addition to increasing waiting time and morbidity^13^ for other patients during trauma team activation.^13^ SO is inconvenient for patients and families^12^ and imposes significant costs on them and caregivers.^14,15^ Various studies have estimated costs ranging from $ 13,294 to $ 67,648 for SO.^6,16^ Thus, SO reduces healthcare efficiency and delays in care delivery. It also increases healthcare costs.^12^ It seems that it is common in low- and middle-income countries due to the lack of standard procedures, imaging, and laboratory technologies in medical centers. In Iranian hospitals like many middle-income countries, unnecessary transfers are considered a weakness of the healthcare system due to poor hospital management, lack of resources, and economic and cultural issues.^17^ However, the rate of SO, its associated factors, and costs due to the lack of a trauma registry system and reliable data have not been examined so far. 

By extracting the data of SO and identifying the effective factors, we can plan, educate and intervene to reduce this procedure. The present study is the first of its kind which was conducted to determine the factors associated with SO in traumatic patients transferred to a HTC in the north of Iran.

## Methods 

***Study Design, Setting, and Participants***

 This cross-sectional study was performed at a level I trauma center in the north of Iran in Poursina Hospital, Rasht. The traumatic patients who had been transferred to this trauma center from LNTCs in Guilan and Mazandaran provinces between 2016 and 2020 were included in the study. All the patients who had been referred for trauma complications (delayed effects due to trauma) (905-909/9), early complications (958), and burns (940-949), were excluded from the study. Moreover, the patients with incomplete records, discharged with personal consent, deemed dead on arrival, and transferred a second time from the receiving hospital were not eligible to enter the study. 

***Data Collection***

Topics under consideration were factors associated with SO and its costs. SO was defined as a situation in which trauma patients transported to a trauma center with ISS (Injury Severity Score) ≤15 were discharged alive within 48 hours of arrival without undergoing any surgical procedure. 

A data entry form was created to collect information. Two Researchers retrieved demographic, clinical, and hospital details of the patients from the health information system of the hospital. Demographic and clinical factors included age, gender, place of incident, mechanism of injury, the injured body region, ISS, reason for transfer, and length of stay (LOS). The ISS was calculated using the Abbreviated Injury Score, where every injury is graded based on severity and topography. The scale ranges from 1 (minor) to 6 (lethal).^18^ The ISS is the sum of the square of the three highest scores of AIS. Patients with an ISS >15 are considered severely injured. Hospital factors for the LNTCs included the name of the hospital and distance to receiving trauma center. The mileage distance was calculated using the shortest road distance between the LNTCs and the trauma center. Also, direct costs paid by the patient (out-of-pocket payment) and hospital (insurance costs and subsidy share) at the receiving trauma center were extracted.

***Statistical Analysis***

The t-test and chi-square test were used to analyze differences in continuous and categorical variables between the two groups, respectively. A multivariate logistic regression estimator was employed to analyze the relationship between demographic, clinical, and hospital factors with SO. All statistical analyses were carried out with Stata Statistical Software (version 14. College Station, TX: StataCorp LP. StataCorp. 2013). P <0.05 was considered significant.

## Results

***Descriptive Analysis***

**Figure 1:**
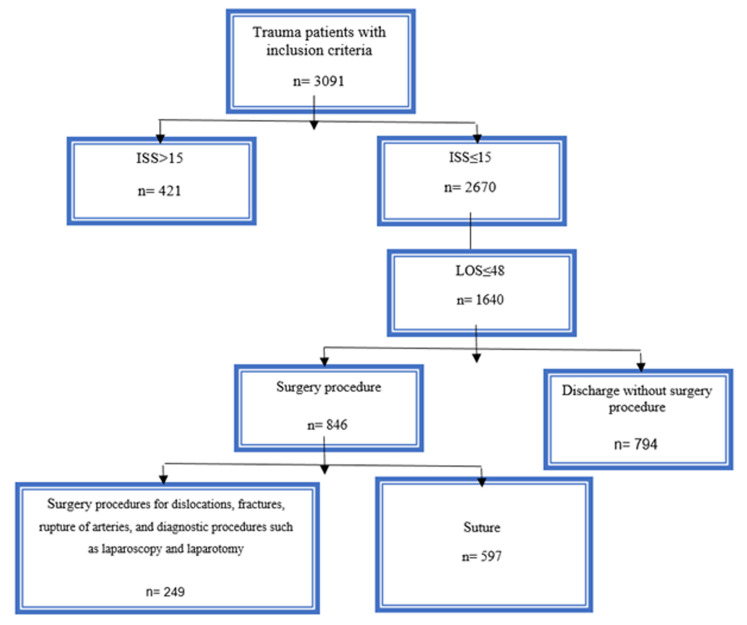
Flowchart of Secondary Overtriage (SO).

During the study period, 3342 trauma patients were transferred to the level I trauma center, 251 (7.51%) of whom were excluded from the study due to lack of inclusion criteria including discharge with personal consent (n= 234), burn (n= 2), and transferring to other hospitals on the first day of hospitalization (n= 15). Therefore, 3091 patients were included in the present study, of whom 794 (25.68%) were identified as SO. The selection process for SO patients is summarized in Figure 1.

Table 1 shows a comparison of the clinical and demographic factors of the SO and appropriate transfer (AT) patients. There is a higher percentage of men in the SO than AT patients (n=541, 68.1% vs n=1848, 80.5%) (P<0.05), and SO patients were younger compared with the AT patients (34-year vs 36-year) (P<0.05). Furthermore, the characteristics and the mechanism of injury of SO and AT patients were different. The mean value of ISS in SO and AT patients was 2 vs. 6 (P<0.05). Head and neck injuries were most common in both groups. Rates of SO and AT were calculated for head and neck injury (n=712, 29% vs. n=1744,71%), face (n=194, 19.3% vs. n=810,80.7%), spine (n=109, 38.2% vs. n=176, 61.8%), thorax (n=76, 27.1% vs. n=204, 72.9%), abdomen and pelvic (n=98, 26.6% vs. n=271, 73.4%), and extremities (n=282, 19.7% vs. 1148, 80.3%), respectively. The difference between the two groups was significant (P<0.05). The lowest SO rate belonged to injuries due to violence (n=30, 3%) whereas motor vehicle collisions (n=613, 24.6%) and falls (n=59, 28.8%) had the highest rate, respectively. Although there was no significant difference between the two groups in mechanism and place of incident, SO patients were more likely to be involved in motor vehicle collisions and falls, which were more common in urban areas. Shortage of neurosurgeons was the most common cause of transmission in both SO and AT patients. However, this amount was more significant in the former than the latter (n=741, 93.3% vs. n=1780, 77.5%, P <0.05). LOS in SO patients was shorter than AT (0.88 vs. 5.33 days, P <0.05).

**Table 1:** Patient-level and Clinical factors for Appropriately Transferred and Secondary Overtriaged patients.

Variable/Subvariable	All (n = 3091)	Transfers Appropriate transfers(n = 2297)	Secondary overtriage(n = 794)	P Value	test
**Age in years, median**	3091(100%)	36	34	**0.008**	**t**
**Sex, n (%)**					
Female	702(22.7%)	449(64%)	253(36%)	**0.000**	**χ2**
Male	2389(77.3%)	1848(77.3%)	541(22.7%)		
**ISS, median**	6	6	2	**0.000**	**t**
**Injured body region, n (%)**					
Head and neck	2456(79%)	1744(71%)	712(29%)		**χ2**
Face	1004(32%)	810(80.7%)	194(19.3%)		
Spine	285(9%)	176(61.8%)	109(38.2%)		
Thorax	280(9.1%)	204(72.9%)	76(27.1%)	**0.000**	
Abdominal and pelvic	369(11%)	271(73.4%)	98(26.6%)		
Upper and lower extremity	1430(46%)	1148(80.3%)	282(19.7%)		
**Mechanism of injury, n (%)**					
Motor vehicle	2489(80.5%)	1876(75.4%)	613(24.6%)		**χ2**
Falls	205(6.6%)	146(71.2%)	59(28.8%)	**0.059**	
Violence	100(3.2%)	70(70%)	30(30%)		
Others1	297(9.7%)	206(69.3%)	91(30.7%)		
**Place of incident**					
Urban	1650(53.4%)	1224(74.2%)	426(25.8%)		**χ2**
Rural	1249(40.4%)	943(75.5%)	306(24.5%)	**0.418**	
Unknown	192(6.2%)	130(67.7%)	62(32.3%)		
**Reason for transfer, n (%)**					
Shortage of neurosurgeons	2521(81.6%)	1780(70.6%)	741(29.4%)	**0.000**	**χ2**
Others2	570(18.4%)	517(90.7%)	53(9.3%)		
**Length of stay in days, median**	4.18	5.33	0.88	**0.000**	**χ2**

1. Other mechanisms of injury including gunshot and animal attack. 2. Other reasons for transfer including lack of orthopedic, general, vascular and reconstructive surgeons or a combination of these.

The distance from the LNTCs to the receiving trauma center was calculated for all transfers; most transferring hospitals were less than 70 km away. Imam Hassan Mojtaba Hospital had the highest number of transfers, which is one of the closest hospitals to receiving trauma centers (n=246, 7.95%). The mean distance between transferring hospitals to this trauma center was equal for SO and AT patients (58.45 and 58.40 KM). There was a significant relationship between SO and AT patients in Valiasr Hospital of Roudbar, Shahid Nourani Hospital of Talesh, and Imam Reza Hospital of Shaft (P <0.05). Valiasr Hospital in Rudbar had the highest level of SO among all hospitals (n=77, 9.69%) ((Table 2 ).

**Table 2:** Appropriateness of Transfer by Transferring Hospitals.

Transferring hospitals	Distance in km	Total n (%)	Appropriate transfer n (%)	Secondary overtriage n (%)	P value compared with other hospitals
Nikookar Hospital in Amlash	76	33(1.06%)	24(72.7%)	9(27.3%)	0.661
Shahid Beheshti Hospital in Astara	173	107(3.46%)	75(70.1%)	32(29.9%)	0.505
Kowsar Hospital in Astaneh	36	245(7.92%)	176(71.8%)	69(28.2%)	0.351
Shahid Beheshti Hospital in Bandar-e Anzali	39	96(3.10%)	68(70.8%)	28(29.2%)	0.727
Shahid Nourani Hospital in Talesh	102	203(6.56%)	157(77.3%)	46(22.7%)	0.009
Shahid Rajaee Hospital in Tonekabon	139	5(0.16)	4(80%)	1(20%)	0.601
Taleghani Hospital in Chalous	195	1(0.03)	1(100%)	0	
Imam sajjad (As) Hospital in Ramsar	113	4(0.12%)	3(75%)	1(25%)	0.802
Salamat Hospital in Rostamabad	53	136(4.39)	90(66.2%)	46(33.8%)	0.429
Valiasr Hospital in Rasht	4	4(0.12%)	2(50%)	2(50%)	0.405
Valiasr Hospital in Roudbar	68	185(5.98%)	108(58.4%)	77(41.6%)	0.001
Shohada Hospital, Rezvanshahr	65	31(1%)	19(61.3%)	12(38.7%)	0.336
Shahid Ansari Hospital in Rudsar	72	143(4.62%)	104(72.7%)	39(27.3%)	0.350
Ghadir Hospital in Siahkal	41	47(1.52%)	28(59.6%)	19(40.4%)	0.148
Imam Reza Hospital, in Shaft	25	101(3.26%)	81(80.2%)	20(19.8%)	0.015
Imam Khomeini Hospital in Sowme'eh Sara	27	202(6.53%)	151(74.8%)	51(25.2%)	0.076
Shohada Hospitalin Tarom	136	6(0.19%)	5(83.3%)	1(16.7%)	0.453
Imam Hassan Mojtaba Hospital, Fouman	30	246(7.95%)	173(70.3%)	73(29.7%)	0.693
22 Aban Hospital and Seyedoshohada Hospital in Lahijan	44	207(6.69%)	133(64.3%)	74(35.7%)	0.106
Amini Hospital in Langarud	59	162(5.24%)	105(64.8%)	57(35.2%)	0.209
Resalat Hospital in Masal	50	167(5.40%)	118(70.7%)	49(29.3%)	0.677
31 Khordad Hospital in Manjil	75	200(6.47%)	127(63.5%)	73(36.5%)	0.068
Unknown	-	560(18.11%)	545(97.3%)	15(2.7%)	-
Distance to Poursina in km, median	58.42	58.40	58.45		0.094

***SO and Associated Factors***

Table 3 shows the results of multivariate logistic regression to identify patient-level, clinical, and hospital-level factors that were significantly associated with SO after adjusting the other variables. At the patient level, sex, and at the clinical level, injured body region (face, abdominal and pelvic, thorax, and spinal injuries), the reason for the transfer, ISS, and LOS were associated with SO (P<0.05). Thus, SO was positively related to the male sex, injured body region, and shortage of neurosurgeons, and negatively related to the ISS and LOS.

**Table 3:** Multivariate Regression Logistic analysis of Secondary Overtriage.

Variable/Subvariable	OR (95% CI)	P Value
**Age in years, median**	0.998(0.989-1.006)	0.697
**Sex, n (%)**		
Female	Ref	-
Male	0.632(0.480-0.832)	0.001
**ISS, median**	0.655(0.0615-0.697)	0.000
**Injured body region, n (%)**		
Head and Neck	1.028(0.586-1.805)	0.921
Face	0.378(0.294-0.485)	0.000
Spine	2.233(1.472-3.388)	0.000
Thorax	1.733(1.131-2.656)	0.011
Abdominal and Pelvic contents	1.463(0.999-2.143)	0.050
Upper and Lower extremity	0.929(0.720-1.199)	0.575
**Mechanism of injury, n (%)**		
Motor vehicle	Ref	-
Falls	0.972(0.616-1.533)	0.903
Violence	0.746(0.421-1.319)	0.314
Others	0.642(0.364-1.131)	0.125
**Place of incident**		
Urban	Ref	
Rural	0.985(0.777-1.248)	0.902
Unknown	-	-
**Reason for transfer, n (%)**		-
shortage of neurosurgeons	2.158(1.185-3.927)	0.012
Others	Ref	-
**Length of stay in days, median**	0.368(0.317-0.428)	0.000
**Distance between transferring hospitals to Poursina in km**	1.003(0.999-1.007)	0.094

Table 4 presents the costs of SO for out-of-pocket payment, the contribution of insurance, and the subsidy, separately. These costs included the cost of accommodation and clinical procedures in receiving trauma center, which averaged 37 $ per patient. The largest percentage of the costs belonged to the insurance contribution.

**Table 4:** Costs of Secondary Overtriage for Transferred Trauma Patients.

	Costs ($)	Average costs ($)	Percent
Out-of-pocket payment	7927	10	27
Insurance	20919	26.6	71.3
Subsidy	488	0.6	1.7
Total	29335	37	100

## Discussion

To the best of our knowledge, the present research is the first study that examined the rate of SO and its related factors and costs in a level 1 trauma center in the north of Iran. SO is a challenge for HTC resources. To improve the consumption of these resources, it is necessary to identify the factors affecting unnecessary transfers to medical facilities in order to take corrective action in the future. Hence, many researchers have studied SO and its related factors.^2,9,10^ Our study suggests that demographic and clinical factors are associated with SO. 

In the present work, the rate of SO in an HTC in Guilan province was calculated to be 25.68%, which is a significant percentage, though other studies have reported higher percentages up to 56 percent,^5,6,19^ or even 70% in pediatric neurosurgical trauma centers. ^20^ Also, there are studies where the amount of SO is lower than the amount calculated in our study.^2,4^ Our results suggest that the male gender is an effective factor in the incidence of SO which can be related to the higher number of traumatic injuries in men. The high rate of trauma in men can be justified by the economic, social, and cultural conditions of society (most women are housewives and more men are involved in outdoor activities).^21,22^ The present findings revealed that ISS and LOS are significantly associated with SO. This means that SO is more common in trauma patients with low injury severity scores, who do not require advanced treatment and clinical procedures in HTCs and are discharged in less than 48 hours if transferred. This result is in line with previous research and confirms their findings.^5,23^

We observed that the injured body region was associated with SO. In the present study, according to previous reports, a high rate of SO was seen in the spine,^2^ chests,^12^ and face^5^ injuries. Considering the odds ratio, spine injuries have the largest share of SO. This finding is justifiable due to the greater frequency of road traffic accidents and falls as injury mechanisms in SO patients. Regarding this finding in addition to the shortage of neurosurgeons as the most common reason for the transmission of patients, it can be stated that hospitals in the province have a lack of neurosurgical services. Studies have shown that 74% of emergency departments had challenges in maintaining on-call coverage, although most problems were related to neurosurgeons (75%), plastic surgeons (81%), and hand surgeons (80%). ^24^ In Ohio, also patients with minor injuries were transferred to higher-level trauma centers of level 3 and non-trauma centers due to a lack of specialties, such as neurosurgery or plastic surgery.^2^ In poor on-call coverage, factors such as low reimbursement of uninsured trauma and emergency patients and a tendency to improve lifestyles by limiting unpredictable on-call hours have been discussed.^25^ Also, the physicians of the transferring facilities due to unavailability of specialist consultation with general surgeons, orthopedic surgeons, and neurosurgeons,^26,27^ are very cautious in dealing with trauma patients, especially with spine, head, and face injuries, and tend to transfer such patients to avoid any consequences and legal responsibilities. However, in previous findings no signs of improvement were observed in the patient's condition after transferring to HTCs, even in the spine, head, and face injuries.^8,28-31^

Although in our study, there was no significant relationship between SO and the distance between the transferring hospitals to receiving trauma centers, it was found that most unnecessary transfers belonged to one of the closest hospitals. LNTCs usually transferred patients with minor injuries to the closest HTC due to various reasons such as lack of clear guidelines, insufficient education, and experience of physicians or healthcare providers in triage, fear of medical outcomes of not transferring patients, and lack of adequate facilities in the center.^2^

Based on the research findings, SO was seen in all LNTCs, but in Rudbar, Talesh, and Shaft hospitals, there was a significant relationship between SO and AT groups. Since these towns are on transit routes, they have a high daily passenger volume, especially on weekends and holidays. As a result, road traffic accidents occur more frequently in these areas, which constitute a high percentage of SO in this study. Triage of minor trauma patients in these hospitals with the shortage of neurosurgical services is a fundamental challenge making the providers refer their patients to advanced trauma centers to avoid any adverse outcomes.

Identifying SO is part of the problem, and developing measures to reduce the number of overtriage is equally important. Adopting appropriate approaches in LNTCs can reduce unnecessary transfers of minor trauma patients.^3^ Developing clearly and sharply outlined guidelines for the care of minor trauma patients can be an effective step. For example, in spine injuries, which as a clinical diagnosis leads to SO, neurologists (when a neurosurgeon is not available) can be summoned to treat and monitor minor trauma patients by expanding the components of guidelines in LNTCs. This method reduces the transfer of patients with low risk and increases the satisfaction of patients and physicians.^2^

In our study, based on the SO flowchart, among the patients who were included in the AT group due to surgical procedures, most cases were assigned as wound management and soft tissue sutures that could be done by a general practitioner in transferring hospitals. Lack of experience and fear of medical consequences for non-transfer of minor trauma patients by ED physicians in LNTCs encourages such transfers. Therefore, it is possible to improve SO by training physicians or healthcare providers of LNTCs, as well as enhancing collaboration between these centers and HTCs. Using telemedicine as a potential solution can help ameliorate the problem and reduce the number of secondary overtriaged cases. It is believed that the cooperation between HTCs and LNTCs using telemedicine leads to improved outcomes, reduced LOS and transfer rates between medical centers, reduced costs of trauma care, and improved access to care in LNTCs.^2,30^ In various studies, telemedicine has been introduced as an approved method for managing trauma patients with different traumas.^32-35^ In one research, establishing emergency transfer coordination centers was presented as an effective solution for managing secondary overtriage cases so that transfers evaluated by this center have a lower risk of being attributed as SO. ^36^

In our study, more than one-third of SO patients had no health insurance, and treatment costs were paid out of pocket. In road traffic injured cases, the costs of eligible people were paid based on Article 92 of Iran’s Law of the Fourth Economic, Social and Cultural Development. In the remaining cases, the costs were covered by insurance companies and subsidies. The costs of SO in previous reports ranged from $ 13,294^16^ to $ 67,512^6^ per patient, which increased annually. In addition, the costs imposed on the patient at LNTCs and the costs of transfer between centers can also be significant. Bukur et al. calculated the average transportation costs between centers separately to be more than $ 40,000 and stated that this amount alone was more than the cost of care in trauma centers. Many of the patients who are unnecessarily transferred to other centers face socioeconomic inequalities such as lack of insurance, and these transfers can impose an additional financial burden on not only the patients and families but also on the healthcare system, contributing to dissatisfaction and uncertainty toward the system, especially if no services are provided after the transfer^6^

**Limitations**

Our study has several limitations. Firstly, like other studies with a retrospective design, examining other effective factors was not possible such as the experiences and awareness of healthcare providers and the quality of care in transferring hospitals. Secondly, we had insufficient information on details before the transfer of patients that could influence the transfer decision. The details about the resources and costs spent on transferring hospitals, the ambulance, and transfer costs were also not available. Due to the lack of a trauma registry system, it is not possible to access and examine these data. Despite these limitations, one of the strengths of our study is that Poursina Hospital is the only level 1 trauma center in Guilan province that provides emergency services for trauma patients 24/7, and other LNTCs transfer their patients to this center, so it can be an appropriate center to review and evaluate the condition of transferred patients.

## Conclusion

About a quarter of trauma patients who were transferred to level 1 trauma centers were identified as SO. This significant percentage of minor trauma patients were discharged from the trauma center alive within 48 hours of admission without undergoing any surgical procedure. Male gender, spine, thorax, and face injuries along with the shortage of neurosurgeons were identified as effective factors in secondary overtriage leading to wastes of the resources of HTCs and huge costs for patients and centers plus the healthcare systems. Holding continuous education courses on the management of trauma patients for medical staff, improving protocols for the transfer of trauma patients, and extending collaboration between HTCs and LNTCs using telemedicine can provide additional guidance during patient transfer for low-level or non-trauma centers. Thus, it reduces the amount of secondary overtriage and the financial and supportive burden on patients, physicians, and hospitals. Given that this is the first research conducted on SO in Guilan province, it is suggested that future studies consider the abovementioned limitations.

**Acknowledgments**

The authors would like to offer their sincerest gratitude to the Iran University of Medical Sciences for their financial support and Ms. Fatemeh Javadi at Allameh Tabataba'i University, Tehran and Guilan Road Trauma Research Center, Rasht for editing the language of the manuscript.
